# A multistep approach to single nucleotide polymorphism–set analysis: an evaluation of power and type I error of gene-based tests of association after pathway-based association tests

**DOI:** 10.1186/s12919-016-0055-4

**Published:** 2016-10-18

**Authors:** Alessandra Valcarcel, Kelsey Grinde, Kaitlyn Cook, Alden Green, Nathan Tintle

**Affiliations:** 1 Department of Statistics, University of Connecticut, 2390 Alumni Drive, Storrs, CT 06269 USA; 2 Department of Biostatistics, University of Washington, NE Pacific St, Seattle, WA 98195 USA; 3 Department of Mathematics and Statistics, Carleton College, 1 N College St, Northfield, MN 55057 USA; 4 Department of Statistics, Harvard University, Massachusetts Hall, Cambridge, MA 02138 USA; 5 Department of Mathematics, Statistics and Computer Science, Dordt College, 498 4th Ave. NE, Dordt College, Sioux Center, IA 51250 USA

## Abstract

The aggregation of functionally associated variants given a priori biological information can aid in the discovery of rare variants associated with complex diseases. Many methods exist that aggregate rare variants into a set and compute a single *p* value summarizing association between the set of rare variants and a phenotype of interest. These methods are often called gene-based, rare variant tests of association because the variants in the set are often all contained within the same gene. A reasonable extension of these approaches involves aggregating variants across an even larger set of variants (eg, all variants contained in genes within a pathway). Testing sets of variants such as pathways for association with a disease phenotype reduces multiple testing penalties, may increase power, and allows for straightforward biological interpretation. However, a significant variant-set association test does not indicate precisely which variants contained within that set are causal. Because pathways often contain many variants, it may be helpful to follow-up significant pathway tests by conducting gene-based tests on each gene in that pathway to narrow in on the region of causal variants. In this paper, we propose such a multistep approach for variant-set analysis that can also account for covariates and complex pedigree structure. We demonstrate this approach on simulated phenotypes from Genetic Analysis Workshop 19. We find generally better power for the multistep approach when compared to a more conventional, single-step approach that simply runs gene-based tests of association on each gene across the genome. Further work is necessary to evaluate the multistep approach on different data sets with different characteristics.

## Background

As next-generation sequencing prices decrease, the demand for statistical methods to interpret the wealth of new data generated by this technology has risen dramatically. The field is now moving past single-marker tests of association for common variants using genome-wide association studies, instead expanding the search to include rare variants. The analysis of rare variants presents unique statistical issues, such as very low power and high multiple testing penalties for single-marker association tests. In recent years numerous methods have been developed that aggregate rare variants into sets, often genes, and run a gene-based test of association to compute a single *p* value for the entire gene. These methods reduce multiple testing penalties and have been shown to increase power in detection of rare variants [1–4].

Gene-based tests of association, while successful in identification of genes with moderate to strong effect sizes, cannot always detect genes with weak effect sizes. Because of multiple testing penalties and lower power to detect genes with weak effects, these genes will often go undetected [5]. By aggregating genes into functionally associated gene sets, or biological pathways, we can reduce the number of tests needed to analyze all the information and, thus, decrease multiple testing penalties. Testing pathways for association with a phenotype of interest may also increase power by aggregating independent associations across a set of genes. In addition, the pathway-based association testing approach allows for the incorporation of biological information and a straightforward, biological interpretation.

Traditional pathway-based association testing involves 2 stages: (a) generate statistics for each gene in a pathway, and (b) aggregate separate gene-level statistics into a single statistic for the pathway [6]. More recently, single-stage association tests have been developed. These approaches are similar to gene-based tests of association in that they group variants into sets and compute a single statistic for the entire pathway. Some groups have considered direct application of tests originally designed as gene-based tests as single-stage pathway tests [6]. Prior research suggests that single-and 2-stage pathway-based association tests will each be optimal under distinct genetic architectures [6].

Although pathway-based association tests can offer improvements in power for detecting association with phenotypes of interest, a significant pathway-based association test does not indicate which of the variants or genes within that pathway are associated with the disease status. Recently, Juraeva et al. [5] proposed a multistep approach for variant-set analysis to address this limitation (first Global Test [7] to identify pathways, then FORGE [Functional element Overlap analysis of the Results of Genome Wide Association Study Experiments] [8] to identify potentially causal genes). However, the Juraeva et al. method has some limitations: (a) it requires a replication data set, (b) is limited in the choice of test statistic at the gene or pathway level, and (c) is not directly applicable to family-based studies. Family-based studies have many advantages, including potentially increased power in rare variant identification [9]. Unfortunately, most pathway-based association methods are unable to directly account for complex pedigrees [10, 11] or cannot account for covariates [9, 12].

In this paper, we propose a novel approach to address existing limitations. In particular, we propose a multistep pathway-based association test for family-based data on complex pedigrees. For the first step we apply a single-stage pathway-based test of association and for the second step we apply commonly-used gene-based test of association to each gene in a significant pathway. We compare our proposed multistep approach to the performance of two commonly used gene-based tests of association (also adapted for family data). In particular, we evaluate the power and type I error of the single- versus multistep association testing approaches using simulated data on blood pressure from Genetic Analysis Workshop 19 (GAW19). We show that our proposed multistep testing approach offers improvements in power, further motivating the application of pathway-based tests of association.

## Methods

### Data description

GAW19 provided genome sequence data (for odd-numbered autosomes), simulated phenotypes, and covariates for 849 individuals from large Mexican American families [13]. Systolic blood pressure (SBP), diastolic blood pressure (DBP), hypertension status, sex, age, smoking habits, and blood pressure medication information is provided at each of 3 examination periods in the simulated data. The simulated data was used in this analysis in order to evaluate type I error and power. To account for the effect of blood pressure medication on blood pressure levels, we made a standard adjustment by adding 10 mmHg and 5 mmHg to the SBP and DBP measurements, respectively, for subjects taking blood pressure medication at each examination [14, 15]. We also calculated the mean arterial pressure (MAP), our response variable of interest, for each individual using the standard formula (MAP = 2/3 DBP + 1/3 SBP). To collapse longitudinal phenotype and covariate information, average age, SBP, DBP, and MAP were calculated and smoking habit was collapsed into a binary variable for whether the subject smoked at least once during the 3 examination periods.

### Creation of sets of variants (genes and pathways)

Variants were mapped to 10,090 genes using a custom version of ANNOVAR (Annotate Variation) [16]. Genes were randomly assigned to 1 of 1346 mutually exclusive sets of genes, which we treat as pathways. These synthetic pathways vary in size and percent of genes that contain at least 1 causal variant. In particular, 82 pathways contain at least 1 of the 245 causal genes. These 82 pathways and 245 genes are the focus of our analysis. Table 1 has more details.

**Table 1:** Settings for creation of synthetic pathways

Number of genes	5				10				Total
Percent Causal	20	40	60	100	20	40	60	100	
Number of pathways	15	11	10	5	15	11	10	5	82

Genes were randomly sampled without replacement and placed into sets of genes, or pathways, of varying sizes. The percent of genes in the pathway containing at least 1 causal variant also varied across sets

### Statistical tests

We identified 2 commonly used gene-based tests of association [17] that account for complex pedigree structure, a quantitative response variable, and are easily implemented using a published R package. One of these methods is a sequence kernel association test (SKAT)–like, or variance component, test of association and the other method is a burden test in the spirit of Combined Multivariate and Collapsing (CMC). Hereafter, we refer to these methods as *VC*_*test*_ and *Burden*_*test*_. *VC*_*test*_ is a weighted sum of single-variant score tests computed as ∑_*j* = 1_^*m*^ *w*_*j*_ *U*_*j*_^2^, where *j = 1,…, m* is an index across m variants, *w*_*j*_ is a weight, and *U*_*j*_ is the score statistic for the association between phenotype and variant *j*. The *skatMeta* function was used with default (beta) weights. *Burden*_*tes*t_ regresses the phenotype on a weighted sum of genotypes within each set by computing ∑_*j* = 1_^*m*^ *w*_*j*_ *U*_*j*_. The *burdenMeta* function was used with default weights (*w*_*j*_ = 1). Both *VC*_*test*_ and *Burden*_*test*_ test for association between a quantitative trait and sets of rare variants, while also accounting for covariates. It should be noted that both tests employ a theoretical, rather than empirical, estimation of the kinship matrix.

### Application of tests

We applied *VC*_*test*_ and *Burden*_*test*_ to the GAW19 data in 2 distinct ways.

### Single-step approach

We applied both a SKAT-like [3] *(VC*_*test*_ *)* and a CMC-like [4] *(Burden*_*test*_ *)* to all 245 genes containing at least 1 causal variant as implemented in a publicly available R package [17]. These gene-based approaches test the null hypothesis that no variant contained within the gene is associated with the disease phenotype (in our case, MAP).

### Multistep approach

First, *VC*_*test*_ was applied to each of the 82 pathways, treating the pathways as very large sets of variants to test if the pathway shows evidence that at least 1 variant is associated with the phenotype. For each pathway flagged as significant, we then apply *VC*_*test*_ to each gene within the pathway, separately, in order to identify significant genes within the pathway. This combination of tests is referred to in the remainder of the paper as the *VC*_*test*_-*VC*_*test*_ approach. We also looked at 3 other combination of tests: *VC*_*test*_ at the pathway level, followed by *Burden*_*test*_ at the gene level (*VC*_*test*_-*Burden*_*test*_); *Burden*_*test*_ at the pathway level, followed by *VC*_*test*_ at the gene level (*Burden*_*test*_-*VC*_*test*_); and *Burden*_*test*_ at both the pathway and gene level (*Burden*_*test*_ *-Burden*_*test*_).

### Estimation of power and type I error

To get empirical estimates for power and type I error of each method, tests were run on each of the 200 simulated phenotypes provided by the GAW19 organizers. For the single-step approach, power of the gene-based tests on a particular causal gene is estimated as the proportion of the 200 simulated phenotypes for which that gene is significant (significance levels discussed below).

For the multistep approach, we first report a similar empirical estimate for power of the pathway-based tests: the proportion of the 200 simulated phenotypes for which a pathway containing at least 1 causal gene (causal pathway) is significant. We also report the proportion of times a causal gene and the pathway in which it is contained are both significant.

Type I error estimates are calculated very similarly. Estimates are achieved by running tests with the variable Q1 as the response. Q1 was simulated by the workshop organizers so as to not be associated with any genes, so we can estimate type I error as the proportion of the simulated phenotypes for which a gene or pathway is significantly associated with Q1.

Initially, we evaluate significance using conventional genome-wide association study alpha levels that penalize for multiple testing: 10^−6^ for single-step approach gene-based tests, 5 × 10^−4^ for multistep approach pathway tests, and 0.05 divided by the number of genes within each pathway for multistep approach gene-based tests. Because of very low observed power with these conservative significance levels (see “Results” below for details), we also consider more liberal alpha levels (0.005 for the single-step approach gene tests, 0.05 for multistep approach pathway tests, and 0.05 for multistep approach gene tests) to facilitate identification of causal variant-sets.

## Results

### Single-step approach: power and type I error of gene-based approach

Using a conventional genome-wide association study alpha level that corrects for multiple testing across all genes (10^−6^), we find that both *VC*_*test*_ and *Burden*_*test*_ are severely underpowered on this data set. Only 1 gene, *MAP4*, which has the strongest effects on SBP and DBP in the GAW19 simulated data, has reasonable power (0.7), and nearly all other genes containing causal variants have little to no power (detailed results not provided). When a more liberal alpha level is used (0.005), power increased above the nominal type I error rate for many genes (detailed results not shown). The top 2 most powerful genes for *VC*_*test*_ were *MAP4* (*VC*_*test*_ and *Burden*_*test*_ power = 1) and *SCAP* (*VC*_*test*_ power = 0.97, *Burden*_*test*_ *power* = 0), while the top 2 hits for *Burden*_*test*_ were *MAP4* and *FLT3* (*Burden*_*test*_ power = 0.31, *VC*_*test*_ power = 0). Modest power for these and a handful of other genes containing causal variants was not at the expense of control of the type I error rate. In particular, Q1 (a simulated variable not associated with genotypes useful for assessing type I error rates) yielded a well-controlled type I error rate (*VC*_*test*_ Mean = 0.003; SD = 0.004; *Burden*_*test*_ Mean = 0.006, SD = 0.006) across the 245 genes containing at least 1 causal variant.

### Multistep approach: power and type I error of pathway-based approach

#### First step: pathway-based association tests

**Fig. 1:**
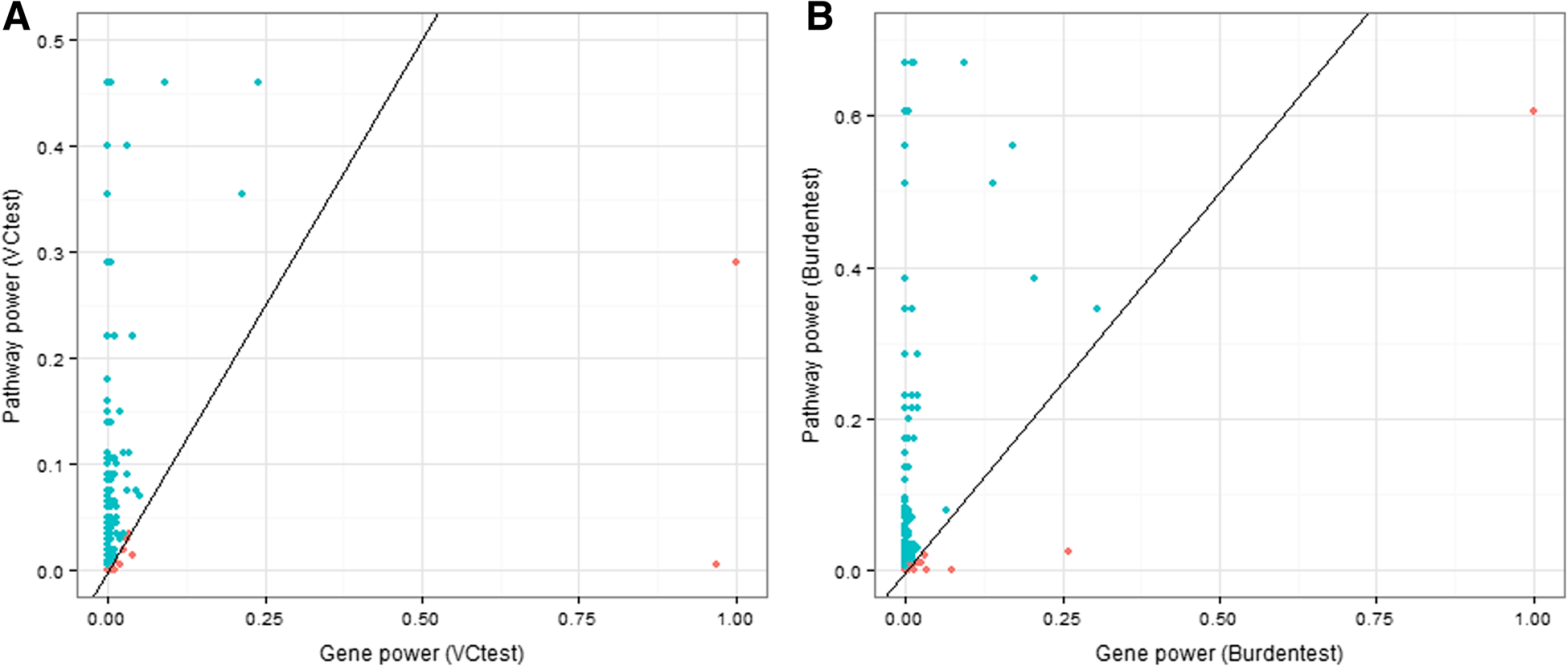
Comparison of power for pathway and gene-based tests. **a**. The power of *VC*_*test*_ for each gene is shown, as well as the power of *VC*_*test*_ for the corresponding pathway that contains that gene. *Blue* dots (above the line y = x) represent genes for which the pathway power is higher than the gene power. **b**. This is the same setup as A, except it shows the power of *Burden*_*test*_ for each gene compared to the power of *Burden*_*test*_ for its corresponding pathway. In general, pathway tests are more powerful than gene-based tests

Both variant-set association tests (*VC*_*test*_ and *Burden*_*test*_) are also severely underpowered at the pathway level when we use a conventional genome-wide association study alpha level (5 × 10^−4^) (detailed results not provided). When a more liberal alpha level is used (0.05), power increased dramatically, and type I error was controlled at the nominal level (detailed results not shown). The top hits are a pathway of 10 genes, 6 of which are causal (*Burden*_*test*_ power = 0.67); a pathway of 5 genes, all causal and containing *MAP4* (*Burden*_*test*_ power = 0.605); a pathway of 5 genes, 3 causal (*Burden*_*test*_ = 0.51); and a pathway with 10 genes, 6 causal (*VC*_*test*_ power = 0.46). When considering the likelihood that a gene would be contained within a pathway determined to be significant, most genes would be at least as likely to be identified if a pathway based test of association was used (*Burden*_*test*_: 94.3 % = 231/245; *VC*_*test*_: 97.1 % = 238/245), with average gains of 9.1 % percentage points *(Burden*_*test*_ *)* and 5.6 % *(VC*_*test*_ *)*, respectively. Figure 1 plots the power of gene-based tests of association versus power of the pathway test for each of the 245 genes.

Power comparisons in the previous paragraph benefit both from a larger significance level (0.05 vs. 0.005) and some lack of granularity in what the results tell the researcher (pathway significance vs. gene significance). In particular, Step 1 does not indicate which variant (s)/gene (s) are significant within the pathway. In the next section, we explore power results considering Step 2 of the multistep approach.

#### Second step: gene-based association tests

**Fig. 2:**
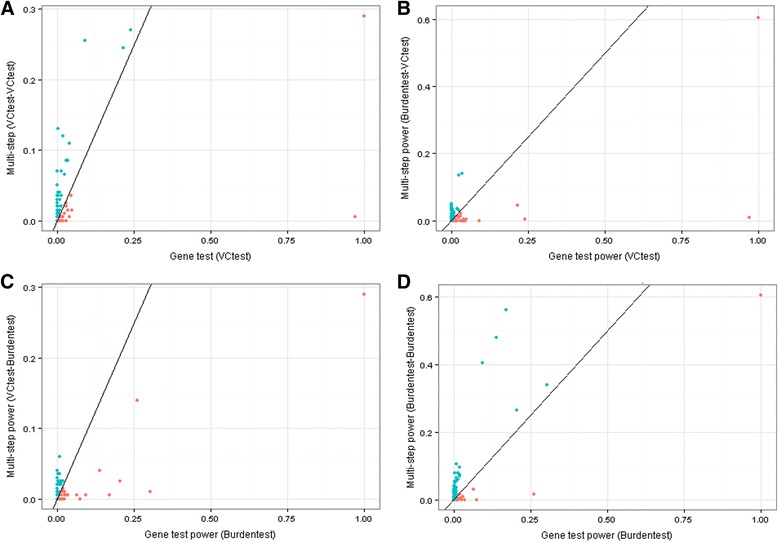
Comparison of power for multistep and conventional approaches. **a**. Comparison of power at each gene for multistep *VC*_*test*_-*VC*_*test*_ versus the single-step *VC*_*test*_ approach, with significance evaluated at alpha levels of 0.05 and 0.005, respectively. The *red* points are genes for which the single-step approach has higher power (below the line y = x) and the *blue* points are instances where our multistep approach has higher power (above the line y = x). There are quite a few instances when the multistep approach offers relatively large improvements in power compared to the single-step gene-based test. **b**. Comparison of power for *Burden*_*test*_- *VC*_*test*_ versus *VC*_*test*_. **c**. Comparison of power for *VC*_*test*_-*Burden*_*test*_ vs. *Burden*_*test*_. **d**. Comparison of power for *Burden*_*test*_-*Burden*_*test*_ versus *Burden*_*test*_. Notice again that there are many instances where the multistep approach offers improvements in power over the single-step gene-based test. Overall we see that our method outperforms the single-step tests by a larger amount, on average, when the first and second step are consistent (*VC*_*test*_-*VC*_*test*_ and *Burden*_*test-*_ *Burden*_*test*_)

A more direct power comparison involves comparison of the joint power (both Step 1 [pathway] and Step 2 [gene] are significant) of the pathway test with a gene-based test, computed only when the pathway test is significant (and using a significance level of 0.05). Even with this consideration, for many genes, the multistep approach offers higher power than conventional single-step gene-based tests. For example, 206 of 245 genes (84.1 %) show power at least as good with the multistep approach as compared to the single-step approach (average power gains of 1.2 %) when comparing the *Burden*_*test*_-*Burden*_*test*_ approach to a single-step *Burden*_*test*_ at the gene level. Results were similar for using *VC*_*test*_-*VC*_*test*_ compared to single-step *VC*_*test*_ (210 genes with at least as good of power using the multistep approach (85.7 % of genes), with average power gain of 8.4 %). Figure 2 summarizes the power differences between multistep and single-step approaches across all 245 causal genes. We note that conducting the same test at the second step as was conducted at the first step is generally a more powerful approach (Fig. 2). A type I error analysis showed overly conservative type I errors for the multistep approach.

Table 2 provides the power of different methods for the genes showing the largest power gains (at least 10 percentage points) when using the multistep pathway approach proposed here (details not shown). We note that the power gain for these genes is not typically a result of being in a pathway with another gene that, alone, is very powerful. For example, *EPS8L1* is in a pathway with 4 other genes. All 4 other genes have power less than 1 % for both *Burden*_*test*_ *and VC*_*test*_ conducted at the gene level. The power gain is realized through a combination of multiple causal genes/variants and reduced significance level.

**Table 2:** Comparison of power for genes showing at least a 10 percentage point increase in power for multistep pathway analysis

Gene name	Pathway description		Single-step power		Multi-step power			
	Number of genes	Percent Causal	VC_test_	*Burden*_*test*_	*Burden*_*test*_-*Burdentest*	*Burden*_*test*_-VC_test_	VC_test_-VC_test_	VC_test_-*Burden*_*test*_
*EPS8L1*	5	60 %	0.010	**0.170**	**0.560**	0.015	0.010	0.005
*FLNB*	5	60 %	0.000	**0.140**	**0.480**	0.035	0.035	0.040
*COL5A3*	10	60 %	0.000	**0.095**	**0.405**	0.050	0.020	0.005
*CASP5*	10	60 %	**0.090**	0.000	0.000	0.000	**0.255**	0.000
*SNAPC3*	10	60 %	**0.005**	0.000	0.000	0.000	**0.130**	0.005
*C21orf33*	10	60 %	**0.025**	0.010	0.105	**0.135**	0.010	0.000
*TNN*	5	100 %	**0.035**	0.010	0.055	**0.140**	0.085	0.005

Power for genes, as well as characteristics of the pathway they are contained in. Bolded entries represent the largest power achieved by each of the methods (single-step and multistep). In each of these scenarios, the multistep approach offers anywhere from a 10 to 40 % increase in power over the conventional single-step approaches

## Discussion

We propose a general framework for conducting pathway analysis on family-based data for rare variants. Current pathway-based and gene-based variant-set testing methods are severely underpowered using standard genome-wide association study significance levels on this data set. However, when more liberal significance levels are used, the novel multistep approach proposed here shows generally increased power over conventional single-step testing approaches. We notice even larger improvements in power when we follow up a pathway test with a similar gene-based test (*VC*_*test*_-*VC*_*test*_ and *Burden*_*test*_-*Burden*_*test*_). This makes sense when we consider that in these cases the genetic architecture (eg, signal) being looked for at both stages is the same. We anticipate seeing similar patterns on other data sets with better power and using standard significance levels, though application of the proposed methods to other data sets is necessary to confirm this.

Importantly, if current methods were modified to provide better control of the multistep type I error rate (instead of being overly conservative) it is possible that the difference in the power of 2 methods would be even greater. However, it is important to note that multistep type I error and power will always be less than or equal to single-step type I error if the same significance level is used in both cases. However, typically, different significance levels will be appropriate as the number of genes under consideration is typically more than the number of pathways.

Our current work is limited in a number of ways including: (a) It was evaluated on a single data set with simulated phenotypes, (b) it only uses single-stage pathway association tests (single test of all variants in a pathway instead of testing each gene first, and then aggregating; see Introduction for details), (c) it incorporates common variants instead of excluding them, (d) it uses a theoretical kinship matrix, and (e) it only evaluates method performance using synthetic pathways. These are all areas for further research, but would not be difficult to incorporate into the current, flexible framework of our approach.

Finally, there were a handful of cases where the single-step (gene-based approach) was better than the multistep approach. This was generally when pathways contained very few other causal genes and/or causal genes with small effect sizes. Consideration of multistep methods that are more robust to the inclusion of noncausal genes may help limit potential power loss in these cases. Further work is necessary.

## Conclusions

A novel multistep pathway approach showed improved power versus single-step approaches for most genes across a wide variety of scenarios. Further work is needed to evaluate the robustness of these findings across a wider range of complex disease architecture.
